# Postharvest Application of Black Mustard (*Brassica nigra*) Seed Derivatives in Sweet Cherry Packaging for Rot Control

**DOI:** 10.3390/foods15010161

**Published:** 2026-01-03

**Authors:** Patricia Calvo, M.ª José Rodríguez, Manuel J. Serradilla, Mª Josefa Bernalte

**Affiliations:** 1 Centro de Investigaciones Científicas y Tecnológicas de Extremadura (CICYTEX), Instituto Tecnológico Agroalimentario de Extremadura (INTAEX), Área de Postcosecha, Valorización Vegetal y Nuevas Tecnologías, Avenida Adolfo Suárez s/n, 06007 Badajoz, Spain; mariajose.rodriguezg@juntaex.es (M.J.R.); manuel.serradilla@juntaex.es (M.J.S.); 2 Escuela de Ingenierías Agrarias, Universidad de Extremadura, Avda. Adolfo Suárez s/n, 06007 Badajoz, Spain; bernalte@unex.es

**Keywords:** allyl isothiocyanate, active packaging, food preservation, fruit quality, fruit rot, natural antimicrobials, *Prunus avium*, storage

## Abstract

Packaging is essential for protecting, distributing, and trading fresh fruit. Antimicrobial packaging, which incorporates natural or synthetic bioactive compounds, can inhibit microbial growth, extend shelf life, and reduce reliance on synthetic fungicides. This study aimed to evaluate the effect of allyl isothiocyanate (AITC), released from black mustard seeds, on the quality and fungal development of ‘Burlat’ sweet cherries during postharvest storage under modified atmosphere. The in vitro and in vivo antimicrobial activity of AITC, released from different amounts of mustard seeds in an ‘Inbox’ system, was compared with fludioxonil, a synthetic fungicide authorised for postharvest use on stone fruits in the European Union. The impact of these treatments on weight loss, headspace gas composition, fruit decay, physicochemical and microbiological quality was also analysed. Results showed that AITC inhibited the in vitro growth of *Cladosporium cladosporioides, Monilinia laxa* and *Penicilium expansum*, and significantly reduced *Alternaria alternata*, *Botrytis cinerea*, and *Geotrichum candidum* after 96 h at 25 °C and 99% RH. Treatment with 100 mg of mustard seeds achieved rot control comparable to fludioxonil, while maintaining higher firmness and delaying skin darkening after 28 days. Overall, natural AITC from mustard seeds appears to be a promising alternative for preserving sweet cherry quality.

## 1. Introduction

Sweet cherries are a high-value crop with significant horticultural trade worldwide. Their vibrant colours, enticing aromas, pleasant tastes, and nutritional attributes contribute to popularity. They also contain a high level of health-promoting compounds [1,2]. The Food and Agriculture Organization of the United Nations (FAO) Statistical Database (2023) [3] reports that Spanish sweet cherry production in 2023 was 104.470 tons. Of this, 33% was destined for export Exported fruit often undergoes long and complex handling chains, which can result in substantial quality losses. Consequently, the fruit industry relies heavily on postharvest technologies to maximise economic potential. Postharvest pathogens further threaten this potential by limiting the shelf life of fresh produce. They contribute to quality deterioration, nutrient loss, and reduced market value. Fungi are responsible for most postharvest diseases affecting fresh produce and therefore pose a significant threat to global food quality and safety [4]. In sweet cherries, the most common pathogens include species of the genera *Penicillium*, *Botrytis*, *Rhizopus*, Mucor, *Cladosporium*, *Alternaria*, and *Monilinia* [5,6].

Traditionally, chemical fungicides are the most effective and economic methods for controlling postharvest diseases. In Spain, for the control of sweet cherries grown in conventional agriculture, the use of a chemical postharvest fungicide (Fludioxonil) is authorised. However, this compound acts by contact and has a limited ability to control fruit rots caused by molds, yeasts and bacteria during packaging and subsequently cold storage. Furthermore, there is great concern among consumers about persistent chemical residues in fruit, antimicrobial resistance, and the environmental impact of conventional methods [4]. For these reasons, and due to the search for safer, healthier, and more sustainable foods [7], there is a great interest in the study of natural antimicrobial extracts released inside the packaging or container in the form of volatile compounds that meet strict public acceptance and environmental safety requirements and extend the shelf life of cherries [8,9].

In this context, packaging plays a crucial role in the protection, distribution and commercialization of food. The demand for innovative packaging solutions to extend shelf life and enhance product safety is increasing, driven by global marketing trends and a growing preference for minimally processed products. Active packaging intentionally modifies the environment of packaged food to maintain its safety, sensory properties and quality [10]. Antimicrobial active packaging eliminates or inhibits the growth of microorganisms, thereby extending shelf life [11]. Interest in natural antimicrobial agents is increasing. As a result, much effort is devoted to exploring plant-derived alternatives to synthetic fungicides, as most possess antifungal properties and can suppress disease-causing pathogens [6].

Among these natural compounds, isothiocyanates (ITCs) constitute a large group of bioactive compounds derived from the enzymatic hydrolysis of glucosinolates and are active against a wide range of pathogens affecting food. Among them, one of the most active seems to be allyl isothiocyanate (AITC), which exhibits strong antimicrobial activity against a wide variety of spoilage and pathogenic microorganisms at low concentrations [12,13]. Black mustard seeds can serve as a natural source for the slow release of AITC into the headspace [14], due to the action of the endogenous plant enzyme myrosinase, which reacts with sinigrin, the primary glucosinolate compound present in these seeds, in humid environments [15,16]. Thus, the present study aimed to evaluate the AITC release kinetics from mustard seed and to assess its in vitro and in vivo antifungal activity against the main postharvest pathogens of sweet cherry. It also examined the effects of AITC on the postharvest life and quality attributes of packaged sweet cherries during low-temperature storage.

## 2. Materials and Methods

### 2.1. Materials

Black mustard seeds (*Brassica nigra*), purchased from an online supplier (Gran Velada, Zaragoza, Spain; http://www.granvelada.com/es/content, accessed on 13 January 2025), were previously defatted and ground in a ball mill (Pulverisette 5, Fritsch, Idar-Oberstein, Germany), and the resulting powder was sieved through meshes of different apertures. For all experiments, the coarsest fraction (>500 µm) was selected. Allyl isothiocyanate (AITC; >94% purity by GC) was obtained from Merck KGaA (Darmstadt, Germany). All other chemicals were supplied by Panreac (Barcelona, Spain) and Sigma-Aldrich (St. Louis, MO, USA).

‘Burlat’ sweet cherry (*Prunus avium* L.) fruit was harvested in May 2025 from 12-year-old trees in an experimental orchard at 804 m altitude in Barrado (lat. 40°05′3″ N, long. 5°52′50″ W), Jerte Valley, Cáceres, Spain. Cherries were handpicked at commercial ripeness, defined by the external colour and size uniformity, sorted to remove damaged and shrivelled cherries and promptly transferred to the laboratory for the subsequent experimental assays.

### 2.2. In Vitro Antifungal Activity

Potato Dextrose Agar (PDA) plates were inoculated with 10 µL of a spore suspension (10^5^ spores/mL) of *Botrytis cinerea*, *Alternaria alternata*, *Penicillium expansum*, *Cladosporium cladosporioides*, and *Geotrichum candidum*. For *Monilinia laxa*, instead of a spore suspension, a 6 mm agar plug containing 5-day-old mycelium was placed in the centre of the agar plate. All fungi were from our collection and had been previously identified by the Plant Health Service of Junta Extremadura (Spain). Plates were sealed in hermetic containers (1 L) at 25 °C and 99% relative humidity (RH) with 50, 100, and 150 mg of mustard seeds. The mycelial diameter of each colony was measured after 48 and 96 h, and the reduction in mycelial growth of each target fungus was calculated according to the following equation:

% reduction in growth = (DC − DA)/DC × 100

where DC is the mycelial diameter (mm) in control plates without mustard seeds, and DA is the mycelial diameter (mm) in plates exposed to mustard seeds.

### 2.3. In Vivo Antifungal Activity

Fruits were disinfected by immersion in a solution of sodium hypochlorite (100 ppm), completely air-dried at room temperature, and then they were wounded by a sterilized stainless-steel rod (3 mm wide × 3 mm deep; one wound per fruit). Each wound was inoculated by using 20 μL of spore suspension (10^5^ spores/mL) of *C. cladosporoides*, one of the majority molds identified in sweet cherries [17]. The inoculated fruits were packaged in transparent polyethylene punnets (1500 cm^3^) with twenty sweet cherries per recipient and 50 mg of mustard seeds and stored at 1 °C and 99 ± 1% RH in darkness. Three punnets were randomly sampled at 0, 6, 10, 13, 17 and 20 days of storage. Untreated inoculated fruits, considered as a control, were placed in the same conditions. Concurrently, untreated and inoculated fruits were stored under the same conditions to assess susceptibility to fruit rot (S.D.). In all cases, the percentage of infected fruit was recorded.

### 2.4. Evaluation of AITC Effect on Packaged Sweet Cherries

To avoid introducing factors that could alter the study’s results, fruit subjected to the same preharvest treatments and grown under identical conditions was used. “Burlat” sweet cherries were harvested following commercial maturity recommendations for prolonged postharvest storage. For proper handling, the black mustard seeds were placed in sachets (“Inbox” format). Commercial modified atmosphere packaging (MAP) was carried out at the facilities of the “Agrupación de Cooperativas Valle del Jerte” using Xtend™ MA/MH bags for cherries (StePac L.A. Ltd., Tefen Industrial Park, Tefen, Israel) in the standard 2 kg format for long-distance export. All treatments were packed in these MAP bags, generating six batches corresponding to the following treatments:

− Sweet cherries (Control)
− Fludioxonil (2.5 mL L^−1^ (*v*/*v*) for 5 min; Scholar 230 SC, 230 g a.i. L^−1^; Syngenta, Basel, Switzerland)-treated sweet cherries (T1)
− Sweet cherries + 50 mg of black mustard seeds (“Inbox” format) (T2)
− Sweet cherries + 100 mg of black mustard seeds (“Inbox” format) (T3)
− Fludioxonil-treated sweet cherries + 50 mg of black mustard seeds (“Inbox” format) (T4)
− Fludioxonil-treated sweet cherries + 100 mg of black mustard seeds (“Inbox” format) (T5)

The selection of the different amounts of black mustard was based on previous studies by our research group with positive results [18,19]. Fludioxonil was applied in accordance with the manufacturer’s recommendations.

Once packed, sweet cherries were transported to CICYTEX facilities in a refrigerated vehicle and stored under controlled temperature and humidity conditions (1 ± 1 °C; 90% RH) for up to 30 days in darkness. Fruit quality was evaluated on the day of harvest and weekly throughout storage. For all analyses, 3 packages per treatment and storage date were randomly sampled from the cool room and analysed immediately after cold storage.

#### 2.4.1. Weight Loss, Headspace Gas Composition and Fruit Decay

Weight loss was calculated by weighing each package immediately before storage (day 0) and at each subsequent sampling date, which refers to predetermined days when measurements were taken, as described by Villalobos et al. (2014) [20]. The evolution of CO_2_ and O_2_ concentrations in the package headspace was monitored using a Checkmate 3 headspace gas analyser (PBI Dansensor, Ringsted, Denmark). Fruit spoilage was evaluated visually, and sweet cherries showing visible mould growth were classified as rotten. Results were expressed as the percentage of spoiled fruit relative to the total number of fruits per treatment.

#### 2.4.2. Microbial Counts

In the proposed postharvest trials, to know the effect of the treatments on the microbial population, the counts of mesophilic aerobic bacteria, and moulds and yeasts were determined using plate count agar (PCA) and potato glucose agar (PDA, pH 3. 5), respectively, as described by Villalobos et al. (2017) [21]. PCA plates were incubated at 30 °C for 48 h, whereas PDA plates were incubated at 25 °C for 5 days. For proper counting, plates with 30 to 300 colony-forming units (CFUs) were considered. Microbial counts were expressed in log10 CFU g^−1^ of sweet cherry.

#### 2.4.3. Physicochemical Analysis

Colour attributes (L*, a* and b*) were measured on the surface of 15 sweet cherries for each package using a Konica Minolta Tristimulus colorimeter (Konica Minolta, CR-400, Tokyo, Japan) using the CIELab space. In addition, the hue angle (h*), calculated as arctg (b*/a*) and the chroma (C*), obtained as (a*2 + b*2) 1/2, were determined. Similarly, 15 fruits were taken per package, and firmness was determined in a Stable Micro Systems TAXT2i Texturometer (Stable Micro Systems, Godalming, Inglaterra), using a 3% compression test with a 25 mm diameter plate on the equatorial zone of each cherry at displacement speed of 0.2 mm s^−1^. Results were expressed as N mm^−1^ [22].

Total soluble solids (TSS), titratable acidity (TA), and pH were measured in an independent homogenate obtained from 25 fresh pitted fruits from per package (n = 3), homogenised using an Omni Mixer homogeniser (Omni International, Marietta, GA, USA). TSS were measured with a Pal01 digital refractometer (Atago, Tokyo, Japón) and expressed as °Brix. TA and pH were analysed with a DL50 Graphix titrator (Mettler Toledo, Columbus, OH, USA) and are expressed as g malic acid per 100 g fresh weight (FW).

### 2.5. Statistical Analysis

Results are presented as mean ± standard deviation (SD). Data normality was assessed prior to ANOVA using the Shapiro–Wilk test, and homoscedasticity was checked using the Levene test. For multiple comparisons, Tukey’s post hoc test was applied when data showed normal distribution and homoscedasticity, and Kruskal–Wallis test when data did not show normal distribution and/or homoscedasticity. Statistical significance was defined as *p* < 0.05. Analyses were conducted using XLSTAT-Pro version 201,610 (Addinsoft 2009, París, France).

## 3. Results and Discussion

### 3.1. In Vitro Antifungal Activity

**Figure 1:**
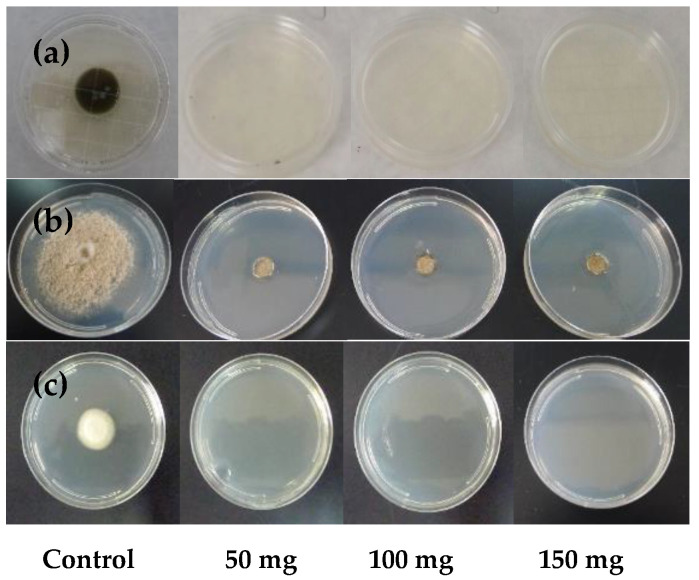
Colony growth of (**a**) *C. cladosporioides*, (**b**) *M. laxa* and (**c**) *P. expansum* on PDA plates incubated for 96 h at 25 °C and 99% RH in the presence of 50, 100, or 150 mg of mustard seed.

**Figure 2:**
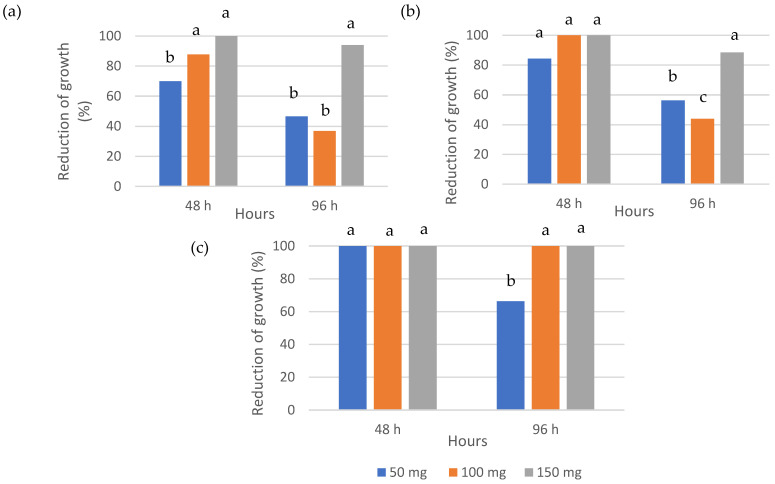
Reduction in growth (%) of (**a**) *A. alternata*, (**b**) *B. cinerea* and (**c**) *G. candidum* on PDA Petri dishes after 96 h at 25 °C and 99% RH, in the presence of 50, 100, and 150 mg of mustard seeds. For each sampling date, significant differences (*p* < 0.05, Tukey’s test) between means are indicated by different letters. Data represent mean values (n = 3).

Data on the in vitro inhibition of mycelial growth of the studied fungi are shown in Figure 1 and Figure 2. After 96 h of incubation, visible growth of *C. cladosporioides*, *M. laxa* and *P. expansum* was observed in the control samples. In contrast, no growth was detected in Petri dishes exposed to 50, 100, or 150 mg of black mustard seeds (Figure 1). The remaining moulds exhibited a different response (Figure 2). For *A. alternata*, after 48 h, the percentage of inhibition increased with dose, reaching approximately 70, 90 and 100% for 50, 100 and 150 mg, respectively. After 96 h, inhibition decreased to about 45% and 35–40% for 50 and 100 mg, respectively, while it remained close to 90–100% at 150 mg. This decrease may be attributed to the tendency of AITC concentrations in the environment to equilibrate after 96 h [23]. A similar trend was observed for *B. cinerea*: at 48 h, inhibition was approximately 85% at 50 mg and 100% at both 100 and 150 mg; at 96 h, inhibition values declined to about 55–60% and 45% for 50 and 100 mg, respectively, but remained close to 90% at 150 mg. In the case of *G. candidum*, all doses resulted in 100% inhibition after 48 h. At 96 h, inhibition at 50 mg decreased to approximately 65–70%, whereas 100 and 150 mg continued to maintain complete inhibition (100%). These results are consistent with previous studies reporting differences in the sensitivity of *P. expansum* and *A. parasiticus* cultured on PDA medium to AITC [24]. In those studies, the different AITC concentrations evaluated caused variable reductions in mycelial growth in Petri dishes, depending on the mold species, with *A. parasiticus* showing greater sensitivity. Furthermore, several authors have reported species-dependent in vitro responses to AITC among both Gram-positive and Gram-negative bacteria associated with food spoilage and foodborne diseases [25,26,27].

### 3.2. In Vivo Antifungal Activity

**Figure 3:**
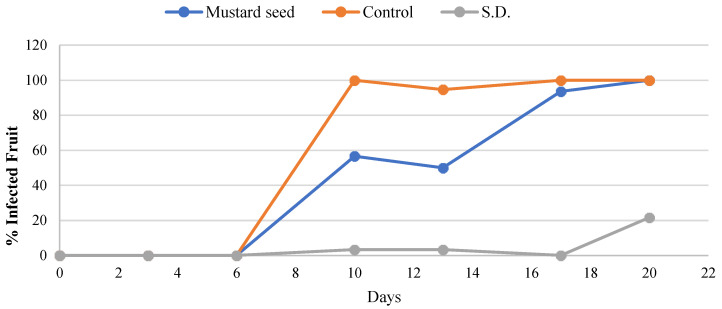
Evolution of the percentage of infected ‘Burlat’ sweet cherries inoculated with *Cladosporium cladosporioides* during cold storage. S.D.: untreated and inoculated fruits used for the susceptibility-to-decay assay. Data represent mean values (n = 3).

In vitro tests provide an initial assessment of the antifungal potential of AITC against postharvest pathogens; however, they should be complemented with in vivo tests to confirm whether the same positive results are obtained. Based on the in vitro results, a treatment with 50 mg of mustard seed was applied. As shown in Figure 3, after 13 days of storage at 1 °C and 99 ± 1% RH, treatment with 50 mg of black mustard seeds significantly reduced the percentage of infected fruit, achieving a 43% inhibition of rot. Nevertheless, after 17 days of storage, no significant differences were observed between treated and control fruits. The antifungal effects of different natural compounds on fruits have been widely studied. Calvo et al. (2021) [19] reported that AITC released from black mustard seeds significantly reduced infection in figs inoculated with *P. expansum*, even at the lowest concentrations tested. More recently, Barea et al. (2024) [18], demonstrated that AITC released from mustard seeds reduced infection rates in tomatoes inoculated with *B. cinerea*. Furthermore, benzyl isothiocyanate (BITC), another natural compound found in cruciferous vegetables, effectively controlled postharvest gray mold in strawberries inoculated with *B. cinerea* [28]. Both AITC and BITC also reduced fungal infections in grapes inoculated with *Aspergillus niger*, *Aspergillus carbonarius* and *Aspergillus ochraceus* compared with controls [29].

### 3.3. Evaluation of AITC Effect on Packaged Sweet Cherries

‘Burlat’ is an early, sweet cherry cultivar notable for its very short postharvest life, which is primarily limited by rapid flesh softening, skin darkening and bruising, stem browning, fruit dehydration, and loss of acidity during storage [30].

#### 3.3.1. Weight Loss, Headspace Gas Composition and Fruit Decay

No significant weight loss (*p* > 0.05) was observed in ‘Burlat’ sweet cherries across treatments during cold storage evolution (Supplementary Figure S1). This result indicates that weight loss was not a critical issue under the experimental conditions evaluated. The lack of significant differences among treatments can be explained by the uniform storage conditions applied, particularly the low temperature and high relative humidity, which play a more decisive role in limiting weight loss than the presence or absence of fungicide. Weight loss in fruit is primarily associated with water loss through transpiration and respiration during storage [31], processes that are substantially reduced under the storage conditions used in this study (1 ± 1 °C; 90% RH). However, other studies on strawberries packaged with AITC or lemongrass oil reported weight losses that varied with cultivar and compound [32,33].

**Figure 4:**
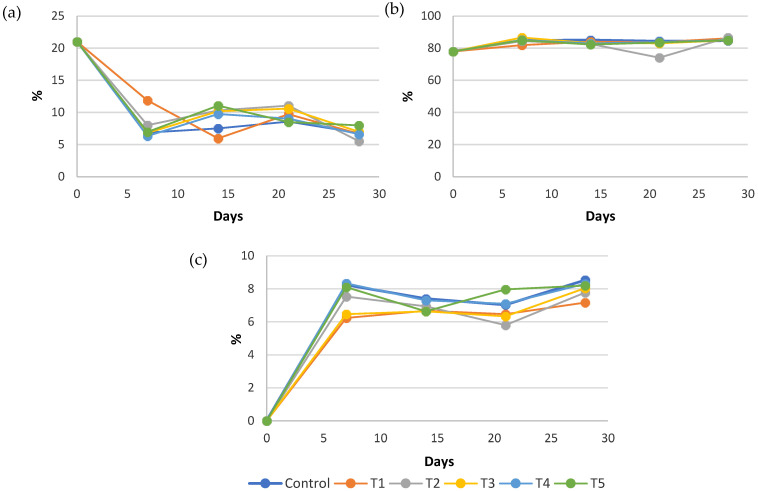
Evolution of (**a**) O_2_, (**b**) N_2_ and (**c**) CO_2_ concentrations in the packaging headspace during cold storage. Data represent mean values (n = 3).

The gas composition in the MAP package headspace is influenced by the respiration and metabolic activity of the sweet cherries. However, no significant differences (*p* > 0.05) in atmospheric composition were observed among the treatments (Figure 4). The gas composition in the MAP package headspace was influenced by the respiration rate and overall metabolic activity of the sweet cherries. As expected, during the initial storage period, the headspace evolved towards an equilibrium atmosphere, characterised by a decrease in O_2_ and a concomitant increase in CO_2_, until a quasi-steady state was reached (Figure 4). However, once this equilibrium was approached, no significant differences (*p* > 0.05) in headspace atmospheric composition were observed among treatments at any given storage assessment time point, as all treatments were sampled and analysed in parallel on the same day for each time point (Figure 4). This limited separation between treatments is likely attributable to the low storage temperature, which would have reduced respiration and thereby constrained treatment-driven divergence in O_2_/CO_2_ dynamics. These results contrast with those reported by Chen et al. (2015) [34] for mulberries stored in rigid plastic containers with snap-on lids, in which AITC reduces fruit respiration. Similar results were reported by Song et al. (2021) [35] for lettuce packed in sealed containers and Kramer et al. (2018) [36] for lettuce and sprouts packaged in plastic bags.

**Figure 5:**
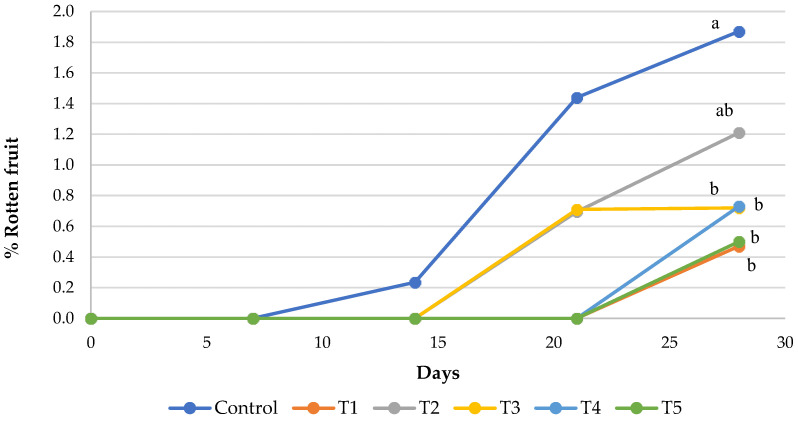
Evolution of the percentage of rotten ‘Burlat’ sweet cherries during cold storage. For each sampling date, significant differences (*p* < 0.05, Tukey’s test) among treatments are indicated by different letters. Data represent mean values (n = 3).

The evolution of fruit rot during cold storage (Figure 5) showed that treatments with black mustard seeds (T2–T5) and the fludioxonil treatment (T1) resulted in lower percentages of rotten fruits than the control. However, only T1, T3, T4, and T5 exhibited significant differences compared to the control (*p* < 0.05). These results indicate that the treatment with 100 mg of mustard seed (T3) controls rot development at a level comparable to Fludioxonil, with a percentage of rotten fruit of 0.7% and 0.5%, respectively, both less than half the percentage calculated in the control treatment (1.9%). Furthermore, no synergistic effect was observed between the phytochemical and AITC.

Wang et al. (2010) [37] also showed that blueberries treated with low AITC concentrations exhibited reduced decay. Similarly, Park et al. (2023) [38] reported that low levels of AITC decrease the incidence of grey mould in blackberries during postharvest storage. Ugolini et al. (2014) [39] further confirmed the possibility of reducing the incidence of postharvest grey mould on strawberries by more than 45% with AITC-based biofumigation. Wu et al. (2015) [40] studied the use of AITC microcapsules for the preservation of mature green tomatoes, showing that the treatment effectively extended storage life and maintained fruit freshness. Likewise, Chen et al. (2022) [41] demonstrated that biofumigation with microbial volatile organic compounds protected postharvest tomatoes from grey mould decay, even after infection with *B. cinerea*.

#### 3.3.2. Microbial Counts

**Figure 6:**
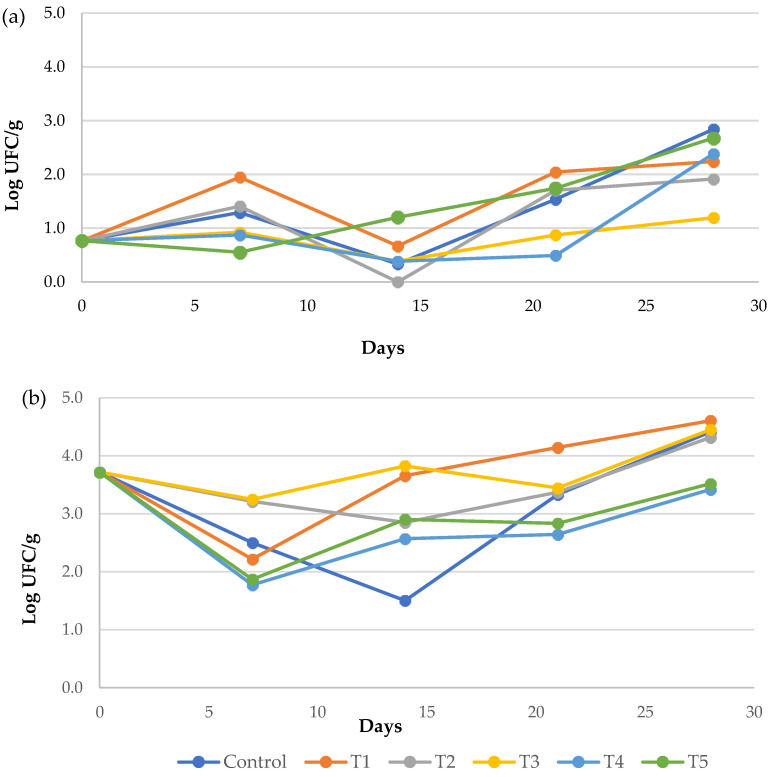
Evolution of (**a**) moulds and yeasts, and (**b**) mesophilic aerobic bacteria in ‘Burlat’ sweet cherries during cold storage. Data represent mean values (n = 3).

Regarding the microbiological count (Figure 6), although no significant differences were observed among treatments (*p* > 0.05), T3 showed the lowest mould and yeast counts. In contrast, for mesophilic aerobic bacteria, T4 and T5 treatments showed the lowest counts, indicating greater effectiveness in controlling their growth.

There is a wide range of inhibition of fungal activity, as supported by several studies that document the effectiveness of AITC in preventing mold growth on food and food processing surfaces [42]. Thus, several authors have investigated the effects of volatile AITC on fresh produce, demonstrating that AITC exhibits microbicidal activity against the native microflora of bean sprouts, fresh-cut iceberg lettuce, and minimally processed shredded cabbage when applied in the package via the gas phase [35,43]. Bahmid et al. (2021) [44] reported that ground meat samples treated with AITC showed lower total bacterial counts than untreated control samples. Similarly, Li et al. (2023) [45] demonstrated that AITC used as a natural antibacterial agent provides a viable strategy for controlling *C. perfringens* in the meat industry.

#### 3.3.3. Physicochemical Characterization

TSS, TA, and pH are key parameters for assessing fruit quality and consumer acceptability. In ‘Burlat’ sweet cherries, the initial values for TSS, TA, and pH were 15.9 ± 0.9 °Brix, 0.59 ± 0.04 g malic acid per 100 g fresh weight and 4.14 ± 0.14, respectively (Table 1). After 28 days of cold storage, TSS values in the control were significantly higher (*p* < 0.05) than in T3 and T4 remaining similar to the initial values. This response may indicate that the respiration rate in control cherries was lower compared to treated fruit, thereby reducing the utilization of sugars as energy substrates. Regarding TA, initial values were significantly higher than those determined after 28 days, and the fludioxonil treatment showed slightly lower values. These results are consistent with Serradilla et al. (2019) [30], who reported a significant reduction in TA in ‘Burlat’ sweet cherries after 30 days of cold storage. Regarding pH, all treatments exhibited a significant increase (*p* < 0.05) after 28 days compared to the initial values. The significant decrease in titratable acidity (TA) and the concomitant increase in pH observed in all treatments are consistent with the metabolism of organic acids during storage, as these compounds are utilized as respiratory substrates. The slight differences among treatments may be associated with variations in respiration rate induced by storage conditions or by the effects of the treatments on fruit metabolic activity. Treatments showing lower TA at the end of storage, particularly those including fludioxonil, promoted greater consumption of organic acids, whereas treatments with intermediate TA values appeared to slow this process.

Regarding colour, the L*, C* and h* colour parameters were significantly higher (*p* < 0.05) in sweet cherries subjected to T3 treatment compared with the control and T1 treatments, indicating that AITC helps prevent the decline of these parameters during storage and preserves the characteristic red colour of freshly harvested fruits. Similar observations were reported by Chen et al. (2015) [34] in mulberry fruits, showing that AITC treatment slowed the senescence process during 15 days of cold storage. Additionally, Kramer et al. (2018) [36] demonstrated that the effect of AITC on fresh produce may be dose-dependent.

Firmness is one of the most commonly used physical parameters for assessing fruit quality [46]. In this study, T3 treatment of sweet cherries exhibited significantly higher firmness values than those treated with T1, with no significant differences compared with the control fruit (*p* > 0.05). These results suggest that AITC treatment may effectively inhibit the softening of cherries observed with fludioxonil treatment. Similarly, Chen et al. (2015) [34] reported that AITC treatment significantly delayed softening in mulberry fruit during cold storage.

Although AITC is associated with beneficial biological activities, it is also known to significantly influence sensory attributes such as pungency, bitterness, and aroma. No sensory analyses were performed in the present study; however, previous work by Barea-Ramos et al. (2024) [18] confirmed that tomatoes packaged with higher amounts of mustard seeds did not exhibit off-flavors.

## 4. Conclusions

The present study demonstrates that the application of AITC, generated from black mustard seeds incorporated into the modified atmosphere packaging of cherries, significantly inhibited or reduced the incidence of postharvest fungal rot. In addition, treated ‘Burlat’ sweet cherries maintained better firmness and showed less colour darkening after 28 days of cold storage than those treated with an approved synthetic fungicide post-harvest. These findings indicate that AITC treatment may slow fruit senescence and serve as an effective alternative for preserving postharvest quality and extending the shelf life of early cherries. In conclusion, this treatment may represent a potential substitute for synthetic fungicides to control rot development in sweet cherries. Nevertheless, further studies are required to determine the optimal dose and to evaluate the effects of AITC not only on the physicochemical and bioactive quality of cherries, but also on sensory attributes and, consequently, consumer perception.

## Figures and Tables

**Table 1:** Mean values of standard quality parameters (TSS, TA, pH, L*, C*, h*, Firmness) of ‘Burlat’ sweet cherries.

	Initial Values (Day 0)	Fruit Quality at 28 Days of Storage					
		Control	T1	T2	T3	T4	T5
TSS	15.9 ± 0.9 ^ab^	17.0 ± 1.10 ^a^	16.4 ± 0.8 ^ab^	16.2 ± 1.4 ^ab^	14.9 ± 0.5 ^bc^	14.3 ± 0.5 ^c^	15.2 ± 1.7 ^abc^
TA	0.59 ± 0.04 ^a^	0.30 ± 0.01 ^b^	0.28 ± 0.03 ^bc^	0.29 ± 0.01 ^b^	0.29 ± 0.01 ^b^	0.26 ± 0.02 ^c^	0.29 ± 0.04 ^bc^
pH	4.14 ± 0.14 ^b^	4.40 ± 0.06 ^a^	4.44 ± 0.08 ^a^	4.38 ± 0.06 ^a^	4.38 ± 0.04 ^a^	4.37 ± 0.07 ^a^	4.42 ± 0.09 ^a^
L*	32.4 ± 2.5 ^a^	27.6 ± 2.3 ^ef^	27.4 ± 2.9 ^f^	28.4 ± 3.6 ^de^	30.3 ± 3.1 ^b^	29.1 ± 2.7 ^c^	29.6 ± 5.0 ^cd^
C*	28.6 ± 6.2 ^a^	18.8 ± 9.5 ^d^	20.7 ± 9.9 ^d^	25.8 ± 11.0 ^bc^	27.5 ± 7.9 ^ab^	25.0 ± 9.3 ^bc^	24.8 ± 11.2 ^c^
h*	17.1 ± 3.0 ^ab^	16.3 ± 3.5 ^bc^	18.8 ± 11.4 ^bc^	17.1 ± 4.2 ^abc^	17.8 ± 3.8 ^a^	15.9 ± 3.3 ^c^	16.9 ± 4.7 ^bc^
Firmness (N/mm)	1.04 ± 0.11 ^ab^	1.08 ± 0.06 ^ab^	0.90 ± 0.03 ^b^	1.11 ± 0.08 ^ab^	1.23 ± 0.09 ^a^	1.10 ± 0.04 ^ab^	1.16 ± 0.19 ^ab^

TSS: Total soluble solids, TA: titratable acidity. Different letters in the same row indicate significant differences (*p* < 0.05) among treatments. Multiple comparisons were performed using Tukey’s test for TSS and firmness; the Games-Howell Test for TA and pH; and the Kruscal-Wallis test for L*, C* and h*.

## Data Availability

Data will be made available upon request.

## Supplementary Materials

The following supporting information can be downloaded at: https://www.mdpi.com/article/10.3390/foods15010161/s1, Supplementary Figure S1. Weight loss during Burlat sweet cherry storage.

## Abbreviations

The following abbreviations are used in this manuscript:

ITCs	Isothiocyanates
AITC	Allyl Isothiocyanate
RH	Relative humidity
PCA	Plate Count Agar
PDA	Potato Glucose Agar
TSS	Total soluble solids
TA	Titratable
