# Supplementary figures and images for: Postharvest Application of Black Mustard (Brassica nigra) Seed Derivatives in Sweet Cherry Packaging for Rot Control
Source: Foods. 2026 Jan 3;15(1):161. doi: 10.3390/foods15010161 (PMC12785422; doi:10.3390/foods15010161)

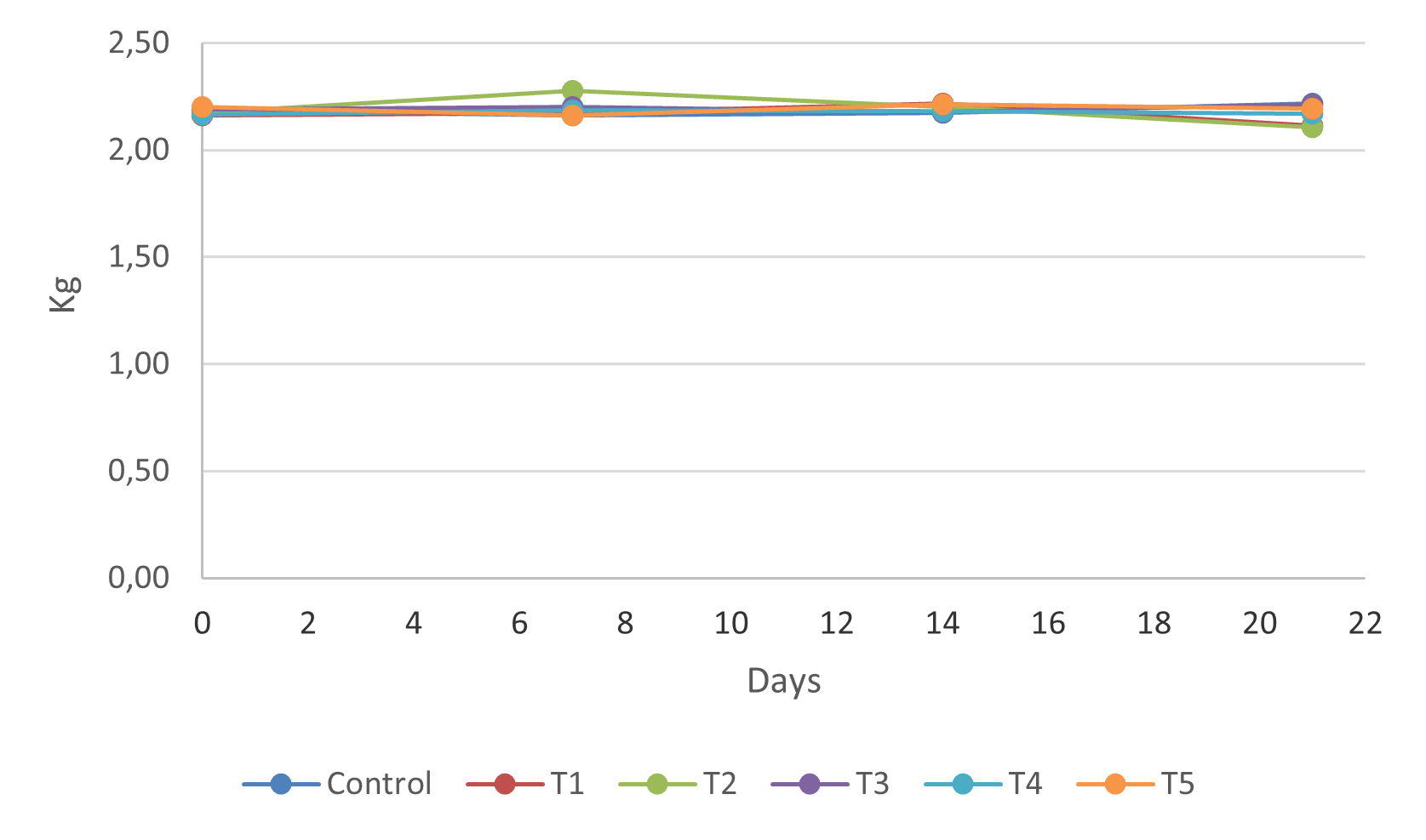

Supplement: Supplementary file 1 [file foods-15-00161-s001.zip › foods-4034545-supplementary.png]
